# Decrypting cancer's spatial code: from single cells to tissue niches

**DOI:** 10.1002/1878-0261.70100

**Published:** 2025-07-25

**Authors:** Cenk Celik, Shi Pan, Eloise Withnell, Hou Wang Lam, Maria Secrier

**Affiliations:** ^1^ Department of Genetics, Evolution and Environment, UCL Genetics Institute University College London UK

**Keywords:** AI, cancer, cell state, cellular niche, digital pathology, geospatial statistics, spatial transcriptomics

## Abstract

Spatial transcriptomics (ST) has emerged as a powerful tool to map gene expression patterns to the local tissue structure in cancer, enabling unprecedented insights into cellular heterogeneity and tumour microenvironments. As the technology matures, developing new, spatially informed analytical frameworks will be essential to fully leverage its potential to elucidate the complex organisation and emerging properties of cancer tissues. Here, we highlight key challenges in cancer spatial transcriptomics, focusing on three emerging topics: (a) defining cell states, (b) delineating cellular niches and (c) integrating spatial data with other modalities that can pave the way towards clinical translation. We discuss multiple analytical approaches that are currently implemented or could be adapted in the future in order to tackle these challenges, including classical biostatistics methods as well as methods inherited from geospatial analytics or artificial intelligence. In the rapidly expanding landscape of ST, such methodologies lay the foundation for biological discoveries that conceptualise cancer as an evolving system of interconnected niches.

## Graphical abstract

Spatial transcriptomics maps gene activity across tissues, offering powerful insights into how cancer cells are organised, switch states and interact with their surroundings. This review outlines emerging computational, artificial intelligence (AI) and geospatial approaches to define cell states, uncover tumour niches and integrate spatial data with digital pathology—laying the groundwork for translating spatial biology into clinical applications.

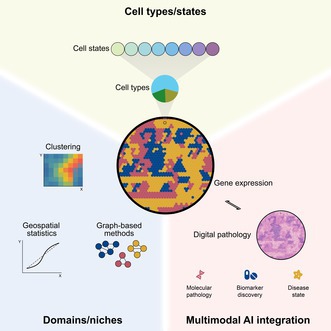

**Abbreviations**
AI: artificial intelligence
CCC: cell–cell communication
EMT: epithelial‐mesenchymal transition
GNN: graph neural network
LR: ligand‐receptor
QC: quality control
scRNA‐seq: single‐cell RNA sequencing
ST: spatial transcriptomics
TME: tumour microenvironment
UMI: unique molecular identifier

## 1 Introduction

Spatial transcriptomics (ST) has recently enabled the profiling of entire tissue sections at minute resolution without disrupting their local architecture, ushering a new era of spatial biology [1]. As we enter this era, new analytical methods will be critical to dissect cell states and relationships across multiple scales. Since its inception, the field has evolved to support unbiased profiling of transcriptomic (Method of the Year 2020 [2]), proteomic (Method of the Year 2024 [3]), genomic and epigenomic landscapes within intact tissues, uncovering systems‐level design principles and regulatory networks beyond the reach of traditional gene‐centric approaches [4, 5]. This revolution, however, brings new computational demands, particularly in cancer, where spatial complexity is extreme. Numerous methods have already been developed to describe cell populations, cell‐to‐cell relationships and domains of interest in cancer spatial data [6, 7, 8], but several challenges remain. Here, we review three key areas of ongoing difficulty in cancer ST analysis: (a) defining cell types and states shaped by both intrinsic (i.e. genomic and epigenetic alterations) and extrinsic factors, within a landscape of profound intra‐ and inter‐tumour heterogeneity; (b) characterising cellular niches and cell–cell interactions, including immunosuppressive dynamics and stromal reprogramming that drive immune evasion and resistance; and (c) integrating ST with other modalities, particularly digital pathology, to map spatially resolved biomarkers, refine tumour classification and advance diagnostic and prognostic applications towards clinical implementation.

## 2 Current analysis workflows in spatial transcriptomics

The advent of high‐throughput transcriptomics and the recent expansion to ST have revolutionised our understanding of biology. Unlike its predecessors, bulk and single‐cell RNA sequencing, ST captures molecular features using a coordinate‐based system. This unbiased approach offers the potential to redefine known and unknown phenotypic characteristics by integrating two modalities: (a) a histological or fluorescence‐based image, which captures tissue morphology and (b) gene expression profiling, which reveals cell types, states and their interactions with the tumour microenvironment (TME) *in situ*.

ST methods can be broadly categorised into two experimental classes: sequencing‐based and imaging‐based platforms [7, 9]. Sequencing‐based methods, such as Visium, Slide‐seq, DBiT‐seq or Stereo‐seq, capture mRNA transcripts via spatially barcoded arrays, high‐definition barcoding, beads or nanoball patterning followed by sequencing [10] (Table 1). These platforms typically generate low‐to‐medium resolution data, with each spot or bead covering multiple cells (1–50 cells depending on tissue context), though Slide‐seqV2 and Visium HD achieve near‐cellular resolution. In contrast, imaging‐based methods (e.g. MERFISH, seqFISH, CosMx or Xenium) use either hybridisation‐based probe detection or multiplexed fluorescence imaging to directly visualise transcripts *in situ*, achieving true single‐cell or subcellular resolution (Table 1). These techniques offer finer spatial granularity but often require more complex image processing, barcode decoding and segmentation workflows [22, 23].

**Table 1:** Overview of key ST platforms, detailing the capture method, spatial resolution, tissue compatibility and key features. Resolution indicates the smallest unit of transcript detection. FFPE compatibility refers to suitability for formalin‐fixed tissue.

Platform	Capture method	Resolution	Tissue type	Remarks	References
Visium	Barcoded slide‐based capture	~ 55 μm (spot diameter)	Fresh frozen, FFPE	Moderate resolution; FFPE version supports curated panels	10x Genomics [11]
Visium HD	Dense barcoded capture on slide	~ 2–5 μm (subcellular)	Fresh frozen, FFPE	Higher resolution version of Visium; whole transcriptome	10x Genomics [11]
Xenium	*In situ* hybridisation with imaging‐based detection	Subcellular (~ 0.2–1 μm)	Fresh frozen, FFPE	Targeted high‐plex *in situ* platform; detects transcripts at near‐single‐molecule resolution; supports curated and custom panels	10x Genomics [11]
CosMx SMI	*In situ* hybridisation + imaging	Subcellular (~ 1 μm)	Fresh frozen, FFPE	High‐plex single‐molecule imaging of RNA/protein	NanoString [12]
GeoMx DSP	ROI‐based UV‐cleaved barcoded probes	Variable (10–600 μm ROI)	Fresh frozen, FFPE	Targeted, high‐plex, region‐specific profiling	NanoString [12]
Slide‐seq/Slide‐seqV2	Bead‐based barcoded array	~ 10 μm (cellular)	Fresh frozen	Higher resolution, technically demanding	Rodrigues et al. [13], Marshall et al. [14]
MERFISH	Multiplexed smFISH with error correction	Subcellular (single molecule)	Fresh frozen, FFPE	Extremely high‐resolution; imaging‐based	Chen et al. [15]
seqFISH/seqFISH+	Sequential hybridisation	Subcellular (~ 0.1–1 μm)	Fresh frozen	Very high gene throughput and spatial resolution	Shah et al. [16], Eng et al. [17]
Stereo‐seq	DNA nanoball arrays with spatial barcodes	~ 500 nm to 100 μm	Fresh frozen	Ultrafine resolution, whole‐transcriptome compatible	Chen et al. [18]
HDST	Barcoded bead array	~ 2 μm	Fresh frozen	Research‐stage, very high‐resolution	Vickovic et al. [19]
DBiT‐seq	Microfluidic deterministic barcoding	~ 10–50 μm	Fresh frozen, FFPE	Multiplexed, supports RNA, protein, epigenome	Liu et al. [20]
Seq‐Scope	Illumina flow cell‐based *in situ* capture	Submicron (< 1 μm)	Fresh frozen	High‐throughput, very high‐resolution	Cho et al. [21]

**Fig. 1:**
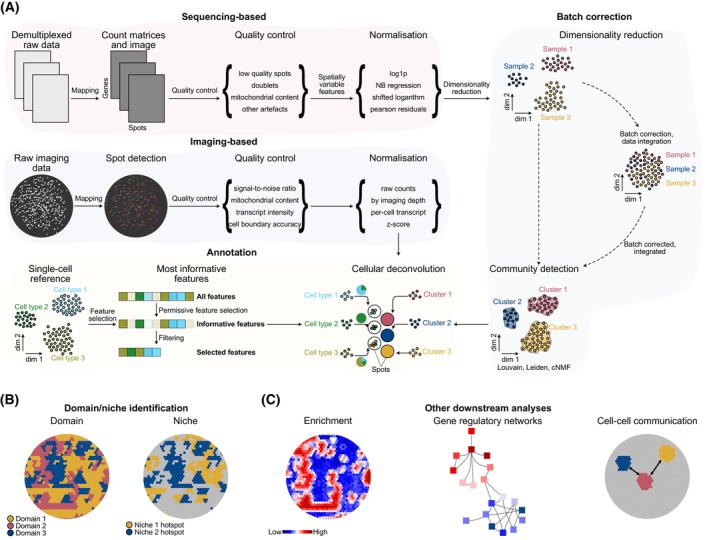
Schematic representation of a spatial transcriptomics workflow, illustrating key steps of downstream analysis. (A) Sequencing‐based: The workflow begins by mapping demultiplexed raw data to a reference genome, generating spatially barcoded gene expression matrices. Next, spots with low‐quality reads and other artefacts, such as high mitochondrial content or doublets, are filtered. An appropriate normalisation method is then applied based on the requirements of the downstream analysis. If working with multiple tissue sections, data integration is performed to harmonise batch effects and identify spots with similar gene expression profiles. Since each spot contains multiple cells, cellular deconvolution is conducted using a well‐annotated single‐cell RNA‐seq dataset as a reference to estimate the proportion of cell types in each spot. Imaging‐based: RNA molecules are captured using multiplexed hybridisation or sequential imaging. Detected transcript spots are localised and aggregated into a count matrix after cell segmentation. The matrix is then normalised (e.g. per‐cell scaling or z‐scoring) for downstream analysis. (B) Niche or domain identification follows, allowing for the interrogation of tumour ecosystems. (C) Additional downstream analyses, such as pathway enrichment, gene regulatory network reconstruction and ligand‐receptor interaction analysis, provide further insights into the tumour biology.

A typical sequencing‐based ST analysis begins by mapping spatially barcoded spots to a reference genome [7]. This demultiplexing step spatially resolves molecular profiles within each spot, generating gene expression count matrices (Fig. 1A). Each spot captures either the gene expression of a single cell or that of a ‘mini’ bulk, typically spanning 1–50 cells depending on the tissue. However, the supercellular resolution of spots is complemented by underlying histological structures, which help link molecular features to tissue morphology. By contrast, imaging‐based ST platforms provide native single‐cell or even subcellular resolution, eliminating the need for deconvolution but introducing critical upstream steps, such as image registration, barcode decoding and cell segmentation (Fig. 1A). Cell segmentation is performed using either nuclear or cytoplasmic markers or by integrating morphological features from co‐captured imaging data to assign transcripts to individual cells. This segmentation step is critical, as accurate attribution of transcripts underpins all downstream analyses. Once transcripts are assigned, a cell‐by‐gene expression matrix is generated, enabling single‐cell resolution analyses akin to single‐cell RNA sequencing (scRNA‐seq) but with spatial context preserved. Subcellular platforms can go further by mapping transcripts to defined intracellular regions (e.g. nucleus, cytoplasm or membrane), enabling investigation of RNA localisation and intracellular gene regulatory processes. Thus, while all ST methods aim to preserve spatial information, higher resolution platforms necessitate additional image‐based preprocessing, segmentation and transcript assignment steps, ultimately enabling more granular insights into tissue architecture and cellular organisation [22].

Quality control (QC) is a crucial step in ST analysis to ensure the accuracy and biological relevance of the data. Low‐quality spots, often characterised by low transcript counts or excessive mitochondrial RNA content, can indicate poor RNA integrity and must be filtered or regressed out. Additionally, spatial artefacts, such as regions with autofluorescence, tissue folding or poor permeabilisation can introduce biases that obscure true biological signals. Spot‐level QC metrics, including the number of detected genes, total unique molecular identifiers (UMIs) and spatial distribution of expression patterns, are routinely assessed to exclude artefactual data (Fig. 1A). Stringent QC steps improve the robustness of downstream analyses and prevent misleading interpretations of spatially resolved molecular features.

Normalisation is essential for mitigating technical variability in ST data, ensuring that observed differences in gene expression reflect biological variation rather than sequencing depth or technical noise. Common normalisation approaches include log transformation, negative binomial, size factor scaling (shifted logarithm) and Pearson residuals, which adjust for variations in sequencing depth across spots (Fig. 1A). However, Bhuva et al. [24] reported that ST raw count data should not be corrected for library size prior to analysis, and scRNA‐seq specific algorithms should be adopted with caution. Seurat's regularised negative binomial regression (SCTransform) method accounts for variation in total mRNA abundance by grouping genes of similar expression levels [25], making it well suited for tissues with differences in cell density. More advanced methods, such as variance stabilisation and quantile normalisation, further refine expression values by addressing spot‐specific technical biases. Appropriate normalisation is thus a critical step that enhances the comparability of gene expression across spatial locations and enables the robust identification of spatial features. In this context, it becomes possible to distinguish spatial domains, which are largely cluster‐based and structurally defined, from spatial niches, which are functionally defined (i.e. by cell states) and may span multiple regions depending on the biological context and microenvironmental interactions.

Although still controversial, batch correction is another critical aspect of ST analysis, particularly when integrating datasets from different experiments, platforms or tissue sections [26]. Variability introduced by differences in sample preparation, sequencing runs or experimental conditions can obscure biological signals. Effective batch correction ensures that spatially resolved gene expression patterns are comparable across samples, enabling robust cross‐condition and multi‐sample analyses. Computational approaches, such as canonical correlation analysis (Seurat [27]), probabilistic models (scVI [28]), hierarchical hidden Markov random field model (STADIA [29]), graph self‐supervised contrastive learning (GraphST [30]) or mutual nearest neighbour correction (MNN [31]) can be applied to align embedding spaces and remove batch effects while preserving biological variation. Count data can also be batch corrected using techniques like negative binomial regression (ComBat [32]) or count‐based regression (Crescendo [33]). A recent benchmarking exercise by Zhang and Hou [34] has highlighted the strengths and remaining challenges of various batch correction approaches, both for same‐platform integration as well as in cross‐platform settings. In fluorescence‐based spatial data, additional considerations, such as correcting for systematic biases in tissue staining or imaging artefacts, may also be necessary.

Following the preprocessing steps, clustering approaches are employed to delineate cellular communities with congruent expression profiles. In parallel, cell annotation using a reference single‐cell dataset or simply based on predefined lists of markers helps define the areas within the cancer tissue that are populated by cancer cells as well as their immune and stromal microenvironment (Fig. 1A). Some ST technologies, such as Visium, measure expression from multiple cells within a single profiled spot, and therefore cellular deconvolution to estimate the cellular composition of ST spots is usually applied at this step [35]. Methods like RCTD [36], Stereoscope [37], Cell2location [38] or Celloscope [39], employing a wide range of techniques from non‐negative matrix factorisation to probabilistic modelling, have been developed to infer the single‐cell contributions to these spatial profiles.

Downstream tasks include cell domain and niche identification, differential expression (Fig. 1B) and enrichment analysis between clusters of interest, and cell–cell interaction analyses (Fig. 1C). Strategies for several of these downstream analytical tasks have been extensively reviewed by Rao et al. [6], Williams et al. [7] and Cheng et al. [22]. As the field matures, challenges beyond these standard analyses start to emerge, in particular with respect to defining distinct cell states, understanding the influence exerted by cells located in close proximity or farther away, and distinguishing correlation and causality in this process.

## 3 Beyond cell type: capturing cell states

**Fig. 2:**
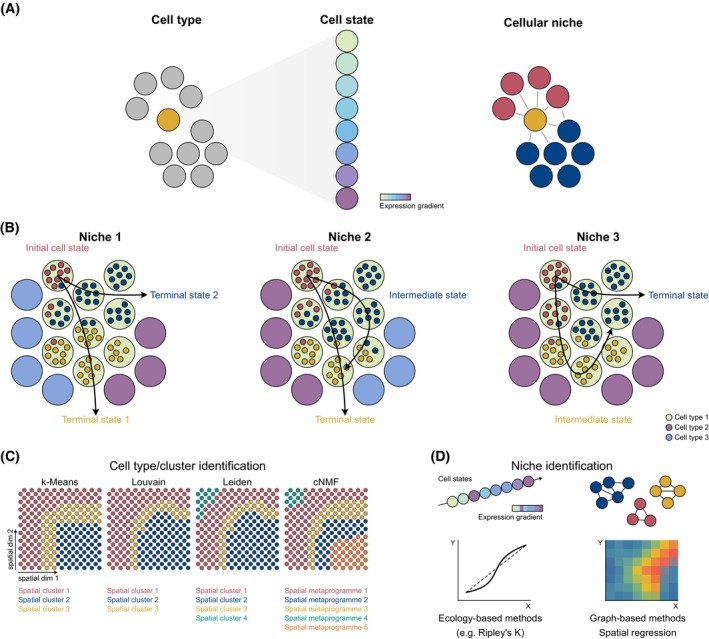
Cell type/state and niche identification in spatial transcriptomics data. (A) Identifying cell types is typically independent of spatial context, whereas cell states are modulated by the surrounding niche through gradient‐based reprogramming of gene expression. (B) Niches are defined as spatial microenvironments composed of multiple interacting cell types whose coordinated behaviour leads to specific functions. Within the tumour microenvironment, cells can exploit their plastic capacity to adapt to different niches. The same cell type can be found in different niches (niche 1, 2 and 3), which may affect their cell state (i.e. initial, intermediate and terminal states). Arrows depict spatial trajectories of cell state transitions, which can also be interpreted as temporal transitions. (C) The plasticity of cells and supercellular nature of spots render traditional clustering algorithms (i.e. k‐means, Louvain, Leiden and cNMF) suboptimal in identifying cell types and states. NMF, Non‐negative Matrix Factorisation. (D) Spatially informed approaches, such as graph‐based methods and ecological models, can capture niche structure and reveal how local microenvironments shape cell behaviour.

Cell‐type identification in ST is key to disentangling the cellular composition of the tissue and enabling the exploration of how cancer cells interact with their environment. Beyond enumerating cell types, however, an even bigger challenge lies in understanding cancer as an ecosystem, that is where the state of the cell is determined by intrinsic factors but also by its neighbours. For this purpose, an essential step after the identification of cell types (e.g. B cell, T cell and cancer cell) is defining cell states (e.g. epithelial‐to‐mesenchymal plasticity, active/exhausted T cell and dedifferentiated cancer cell) in ST data (Fig. 2A).

Unlike discrete cell types, many cellular states exist along a continuum [40, 41], requiring analytical frameworks capable of capturing gradual transitions rather than binary classifications. Advances in single‐cell RNA sequencing have enabled the development of methods that can resolve this continuum with increasing granularity, both experimentally [41] and computationally [42, 43], and these approaches are portable to ST datasets. One widely used strategy involves scoring cells based on predefined gene signatures, using enrichment‐based approaches, such as gene set enrichment analysis [44] to quantify state transitions. Dimensionality reduction techniques, including non‐negative matrix factorisation and principal component analysis, as implemented in tools like ProjectR [45] or more advanced transfer learning methods like scGoGAPS [46], are often employed to extract co‐expressed gene modules and reveal underlying axes of variation. Probabilistic approaches, such as Hidden Markov Models or Gaussian Mixture Models, have also been employed to infer discrete cell states from continuous expression measurements, as applied by our group to study the epithelial‐to‐mesenchymal transition (EMT) [47] or by Liu et al. [48] to identify bimodal gene expression patterns linked with driver events in cancer. Temporal progression of cell states can also be inferred through pseudotime analysis, with tools like SpaceFlow [49] and PSTS [50] reconstructing dynamic trajectories from static scRNA‐seq snapshots.

More recent innovations have extended these concepts further. For example, CELLSTATES [51] refines clustering by partitioning cells into expression states that are statistically indistinguishable, allowing for resolution that respects biological noise while maximising interpretability. Similarly, SeaCells [52] introduces an archetypal analysis‐based method to group individual cells into ‘meta cells’ that retain the core features of gene expression profiles, while minimising information loss. Other methods, such as MARGARET [53], use graph partitioning and pseudotime ordering to infer complex trajectory topologies. Together, these and other similar approaches offer a diverse toolkit for disentangling the complexity of continuous cell state landscapes, and their application to single‐cell data has been extensively reviewed by Trapnell [54], Tirosh and Suva [55] and Rafelski and Theriot [56]. While directly applicable to ST data, these tools nevertheless remain blind to the spatial information offered by this technology.

When it comes to directly capturing cell states in spatially resolved datasets, the field is still in its infancy. Many studies project cell states inferred from single‐cell RNA‐seq onto ST using simple clustering approaches to identify areas within the tissue where cancer cells occupy distinct states, for example as shown by Liu et al. [57], where mapping distinct cell lineages identified perinecrotic immunosuppressive niches in glioblastoma. To address the challenge of mapping discrete cell states in a statistically principled manner, we have employed the Getis‐Ord Gi* statistics to develop SpottedPy [58] and illustrated how it can be used to capture a flexible number of EMT states in cancer tissues. CellCharter [59] leverages Gaussian mixture models to identify stable and morphologically distinct clusters, thereby delineating spatial niches with characteristic shapes and degrees of cellular plasticity. In their study, Varrone et al. [59] demonstrated its ability to capture two co‐existing cancer cell states with distinct microenvironmental interactions in lung adenocarcinoma.

For supercellular ST platforms (i.e. lacking single‐cell resolution), such as 10x Visium or GeoMx, an ongoing challenge is disentangling the origin of the different expression signals as they are profiled from multiple cells co‐located within the same spot or region of interest. Cellular deconvolution methods mentioned in the previous section can help disentangle such signals so that the cell state quantification is then performed for specific cell populations, as shown by us [58, 60] and others [61]. Nevertheless, resolving these signals remains limited, and the rapid pace of advancement in the field will likely bypass the need for such approaches in the near future as most technologies are moving towards single‐cell or subcellular resolution.

Despite these advances, no single metric or method for defining a cell state or cell type is universally accepted, underscoring the ongoing need for standardised criteria and consensus across the field, alongside spatially aware methods or benchmarks to validate these states. Furthermore, challenges remain in modelling spatial continuity and in developing flexible strategies that can capture spatial organisation across multiple scales. Mapping cell types and states introduces a linked challenge, that of defining cellular domains, niches or communities with congruent patterns resulting in emergent functional behaviour (Fig. 2C), which we discuss in the next section.

## 4 Characterising the cancer cell niche

Defining and understanding cellular niches is a new frontier recently enabled by spatial biology. Recent efforts to map cellular communities and cancer hallmarks across different cancer types [62], revealing specialised compartments and local drug sensitivities, illustrate the need to develop robust methods to capture functional niches that could inform both cancer biology and treatment [63]. Below, we outline classical statistical approaches that have been adapted to define a cell niche, alongside emerging trends in using geospatial metrics or AI to explore cell–cell interactions and dependencies at various scales (Table 2).

**Table 2:** Existing tools that can capture cellular niches in ST data. The underlying statistical approach, sample applications, platforms that the methods were originally developed for and link to the respective publication are listed for each method. Tools are listed in alphabetical order.

Tool	Method	Application	Compatible platform	References
BayesSpace	Bayesian modelling, Markov random fields	Spatial domain detection, resolution enhancement	10x Visium	Zhao et al. [64]
CCST	NMF, probabilistic modelling	Cell‐type decomposition, cell‐type proportion estimation	10x Visium	Li et al. [65]
CellCharter	Graph‐based clustering, Gaussian mixture models	Spatial architecture identification, tissue stratification	10x Visium, CosMx	Varrone et al. [59]
conST	Contrastive learning, Graph convolutional networks	Spatial domain detection, data integration, representation learning	10x Visium, Slide‐seq	Zong et al. [66]
DeepST	Deep neural networks, supervised learning	Cross‐section spatial domain prediction, knowledge transfer	10x Visium, Slide‐seq	Long et al. [30]
Giotto	Spatial autocorrelation	Spatial gene identification, cell–cell interaction, enrichment analysis	seqFISH^+^, MERFISH, STARmap, osmFISH, CyCIF	Chen et al. [67]
GraphST	Graph neural networks, autoencoders	Spatial clustering, representation learning	10x Visium, Slide‐seq, Stereo‐seq	Long et al. [30]
Renoir	Spatially aware regression	Spatial ligand activity inference, cell–cell communication	10x Visium	Rao et al. [68]
scNiche	Logistic regression	Spatial niche identification, microenvironment characterisation	10x Visium	Qian et al. [69]
SEDR	Deep autoencoders, variational inference	Spatial domain detection, latent representation learning	10x Visium	Xu et al. [70]
SpaceFlow	Self‐supervised learning, spatial vector field inference	Spatial trajectory inference, developmental/disease gradient modelling	10x Visium	Ren et al. [49]
SpaGCN	Graph convolutional networks	Spatial domain detection, spatial gene identification, image‐informed analysis	10x Visium	Hu et al. [71]
SpottedPy	Getis‐Ord Gi*	Hotspot analysis	10x Visium	Withnell et al. [58]
STAGATE	Graph attention networks, variational autoencoders	Spatial domain detection, heterogeneity analysis	10x Visium	Dong et al. [72]
stMMR	Multimodel regularised regression	Integration of ST with histology, spatial effect modelling	10x Visium	Zhang et al. [73]

### 4.1 Unsupervised and signature‐guided determination of expression domains

Unsupervised clustering of spatially resolved gene expression data is the most straightforward approach to delineating tissue areas with relatively homogeneous expression patterns, which could be considered distinct ‘domains’ or ‘niches’. Methods widely applied in single‐cell RNA‐seq, such as Louvain, Leiden or cNMF leveraging highly variable genes, have been ported to ST and implemented by pioneering tools like Giotto [74], but they effectively discard the spatial information and spatially variable genes and therefore are inadequate to defining spatially distinct *functional* niches (Fig. 2B). Methods like SpaGCN [71], BayesSpace [64], Niche‐DE [75] and COVET [76] are spatially aware and thus allow a more flexible delineation of tissue territories with congruent expression. Tools like NeST [77] and GASTON [78] extend this paradigm by integrating hierarchical or topographical information, enabling the discovery of nested co‐expression domains that often align with known tissue architecture. Other approaches, like Voyager [79] and Monkeybread [80], incorporate prior knowledge in the form of user‐defined gene signatures or cell‐type labels to further refine spatial clustering outcomes, thus allowing a hypothesis‐driven interrogation of the data. Several recent clustering‐based methods have been benchmarked for multiple ST technologies by Yuan et al. [81].

### 4.2 Geospatial statistics

Geostatistical and ecological frameworks are being increasingly adopted in biology to quantitatively assess the spatial organisation of tissue architecture [82] (Fig. 2C). Geospatial metrics, such as Ripley's *K* function—commonly used to distinguish between random and clustered spatial patterns—have been applied to characterise interactions within the TME, including the spatial distribution of immune cells in ovarian cancer [83]. Similarly, point pattern analysis and spatial autocorrelation metrics like Moran's *I* have been used to evaluate the degree of clustering across histological features [84] and address key challenges in spatial data, such as spurious associations due to spatial dependence [82]. Hotspot analysis, a method widely used in fields such as epidemiology and criminology, has also found application in pathology, enabling the identification of immune cell‐enriched regions and supporting patient stratification in breast cancer [85]. However, while these approaches have shown promise in histopathological contexts, their integration into ST remains relatively limited. ATHENA [86] was amongst the early adopters of geospatial metrics for ST, and more recent methods, such as Voyager [79] and MuSpAn [87], have recently been introduced, offering a suite of geostatistical, mathematical and network topology functions specifically adapted for ST data, thereby facilitating broader application of spatial statistics in molecular tissue profiling. However, a key open question in applying geostatistical approaches to spatial tumour biology is determining the appropriate scale at which to define a niche. Our method SpottedPy [58] utilises the Getis‐Ord Gi* geospatial statistic to flexibly explore tumour hotspots at increasing neighbourhood sizes based on user‐defined signatures or annotated gene set signatures (e.g. from MSigDB), thereby enabling the capture of both local and globally stable relationships between cells across the tissue. Using SpottedPy, we have uncovered hotspots delineating the gradual EMT progression of cancer cells across the tissue, pinpointing immune evasive hybrid niches with premigratory features [58, 60]. In the future, we envision there will also be scope for applying geospatial regression approaches to assess cellular dependencies and perform predictions of future cell states based on current states captured in ST slides.

### 4.3 Graph representations and AI

Graph‐based abstraction of gene expression data has become an increasingly prominent approach for analysing ST slides [88]. Combined with AI, it facilitates a wide range of applications, including traditional ST tasks like cellular deconvolution [89], clustering or dimensionality reduction [90], but also more complex downstream analyses, such as inference of autocrine and extracellular gene interactions [91] or modelling of cellular neighbourhoods [65, 92] (Fig. 2D). The concept of representing cells as nodes within a graph structure dates back to early work in cancer pathology in the 2000s, where graph metrics were shown to effectively distinguish between healthy and inflamed or diseased tissues [93]. Recent work has demonstrated that graph neural networks (GNNs) can model tissue‐level emergent phenotypes, such as immune cell dispersion in colorectal tumours [94] or liver injury niches, as demonstrated by scNiche [69]. Alignment‐free integration methods like STAIG [95] employ graph‐contrastive learning to delineate niches aligning with the underlying histology of the tissue. The application of GNNs is continuously expanding in ST, in particular for elucidating cell–cell interactions as explained in the next subsection. Going forward, we envision such methods will be particularly powerful for defining spatial dependencies that can help define a *functional* niche, and not simply one defined by agglomeration of cell types.

### 4.4 Dissecting cell–cell communication within the niche

A niche is ultimately an ecosystem where the cells influence each other's behaviour, and therefore, understanding how cells communicate should be an essential part of defining a niche. Such insights can be obtained through ligand‐receptor (LR) co‐expression or signal transduction inference originally developed for single‐cell data (Fig. 1C). Tools like CellPhoneDB [96], NicheNet [97] or Scriabin [98] exemplify this, while spatially aware methods, such as LIANA+ [99] and CytoTalk [100], extend these capabilities to spatial datasets. These tools have been comprehensively reviewed elsewhere [101, 102]. However, accurately identifying true cell–cell interactions remains challenging, particularly in sequencing‐based ST, where LR annotations are often incomplete or ambiguous [103]. Imaging‐based spatial proteomics may offer more reliable detection in this respect, yet it typically covers a limited panel of proteins, making it less suitable for discovery research [104]. Renoir [68] addresses this gap by integrating either spot‐level ST data with annotated scRNA‐seq from the same tissue or single‐cell resolution ST, to infer spatial neighbourhood activity scores for curated ligand‐target pairs at each spot. Despite its strengths, Renoir's reliance on curated ligand‐target interactions rather than LR pairs, along with accurate annotations, can restrict its capacity to detect *bona fide* or complex interactions. Its neighbourhood‐based model and computational demands may also pose challenges in diffuse tissues or large‐scale datasets.

A growing number of computational tools have emerged to infer spatially resolved cell–cell communication (CCC) from ST data, each leveraging distinct modelling strategies to integrate spatial and biological information (Table 3). These methods range from traditional statistical approaches to cutting‐edge deep learning frameworks. For example, COMMOT [105] applies optimal transport theory to model spatial CCC, whereas TWCOM [113] uses a Tweedie‐based generalised additive model compatible with both spot‐level and single‐cell resolution. Graph neural networks are also increasingly prominent: tools like DeepTalk [106], NEST [108] and SpaTalk [112] employ attention mechanisms or knowledge graphs to capture spatial dependencies and LR signalling dynamics. Meanwhile, NiCo [109] and Renoir [68] integrate spatial data with reference atlases or pathway‐level ligand‐target activity to enhance biological interpretability. Other methods, such as SpaGraphCCI [111] and SpaCCC [110], embed gene expression and image‐derived features or functional networks into latent spaces to infer novel or distal CCC events.

**Table 3:** Existing methods to infer cell–cell interactions. The underlying statistical approach, sample applications, platforms that the methods were originally developed for and link to the respective publication are listed for each method. Tools are listed in alphabetical order.

Tool	Method	Application	Compatible platform	References
COMMOT	Optimal transport	Infers spatial CCC from ST data	10x Visium	Cang et al. [105]
DeepTalk	Graph attention network integrating scRNA‐seq and ST	Dissects spatial CCC in complex biological systems	10x Visium, Slide‐seq	Yang et al. [106]
IGAN	Joint probability density	Captures CCC at single‐cell or spot resolution; explores upstream and downstream pathways of LR interactions	10x Visium	Zhu et al. [107]
NEST	Graph attention network with unsupervised contrastive learning	Detects CCC in spatial and single‐cell transcriptomics data; integrates spatial proximity and signal strength	10x Visium, Slide‐seq	Zohora et al. [108]
NiCo	Integrates ST with scRNA‐seq reference data to infer spatial niche effects	Identifies niche‐specific interactions and cellular state drivers in tissue development and homeostasis	Slide‐seqV2, Slide‐tags, Stereo‐seq	Agrawal et al. [109]
Renoir	Pathway‐level activity of ligand‐target gene sets and domain‐specific ligand‐target activities	Context‐specific CCC interactions and therapeutically relevant cellular crosstalk	10x Visium	Rao et al. [68]
SpaCCC	Fine‐tuned single‐cell LLM and functional gene interaction network to embed LR pairs in latent space	Detects novel LR pairs and patterns using ST; visualises CCC at single‐cell type level	10x Visium, Slide‐seq	Ji et al. [110]
SpaGraphCCI	Deep learning with co‐convolution to integrate gene expression and image features into low‐dimensional space	Infers proximal and distal spatial CCC interactions from multimodal ST data	10x Visium, Slide‐seq	Zhang et al. [111]
SpaTalk	Graph network with knowledge graph; models LR‐target signalling	Infers spatial CCC by integrating single‐cell and ST data	10x Visium, Slide‐seq	Shao et al. [112]
TWCOM	Tweedie distribution‐based model within a generalised additive model framework	Infers CCC from both single‐cell and spot‐based ST data	10x Visium, Slide‐seq	Wu et al. [113]

In addition, emerging AI models are starting to uncover spatial organisation principles and interaction dynamics previously inaccessible through conventional methods. Unsupervised representation learning approaches, such as SEDR [70], combine masked self‐supervised learning with variational graph autoencoders to generate dimensionally reduced representations that capture both expression patterns and spatial context, offering superior clustering performance while effectively imputing missing values. Additionally, cell–cell interaction‐aware frameworks, such as SPACE [114], develop embeddings that simultaneously encode expression profiles and neighbourhood interactions, revealing spatially defined cell subtypes and distinct tissue modules with characteristic intercellular signalling networks.

These tools reflect a growing trend towards the integration of spatial proximity, functional annotations and multimodal data to gain context‐specific insights into the tumour microenvironment. The ST field thus represents a convergence point for techniques spanning computer science, statistics, geography and ecology. Despite these advances, current analytical frameworks still struggle to capture and compare spatial interactions across multiple scales, particularly when examining both short‐range (cell–cell contact) and long‐range (paracrine signalling) interactions between distinct tissue regions or cell populations, with very few existing tools (e.g. ATHENA [86]) providing metrics for this purpose. This limitation is especially critical in cancer research, where spatial boundaries of key processes, such as hypoxia, remain poorly defined. As such, frameworks that focus exclusively on local neighbourhoods risk overlooking broader spatial dependencies that underpin tumour progression. Future tools should therefore prioritise scalable models that enable multiscale analyses of spatial interactions.

## 5 Integrating spatial transcriptomics and digital pathology

**Fig. 3:**
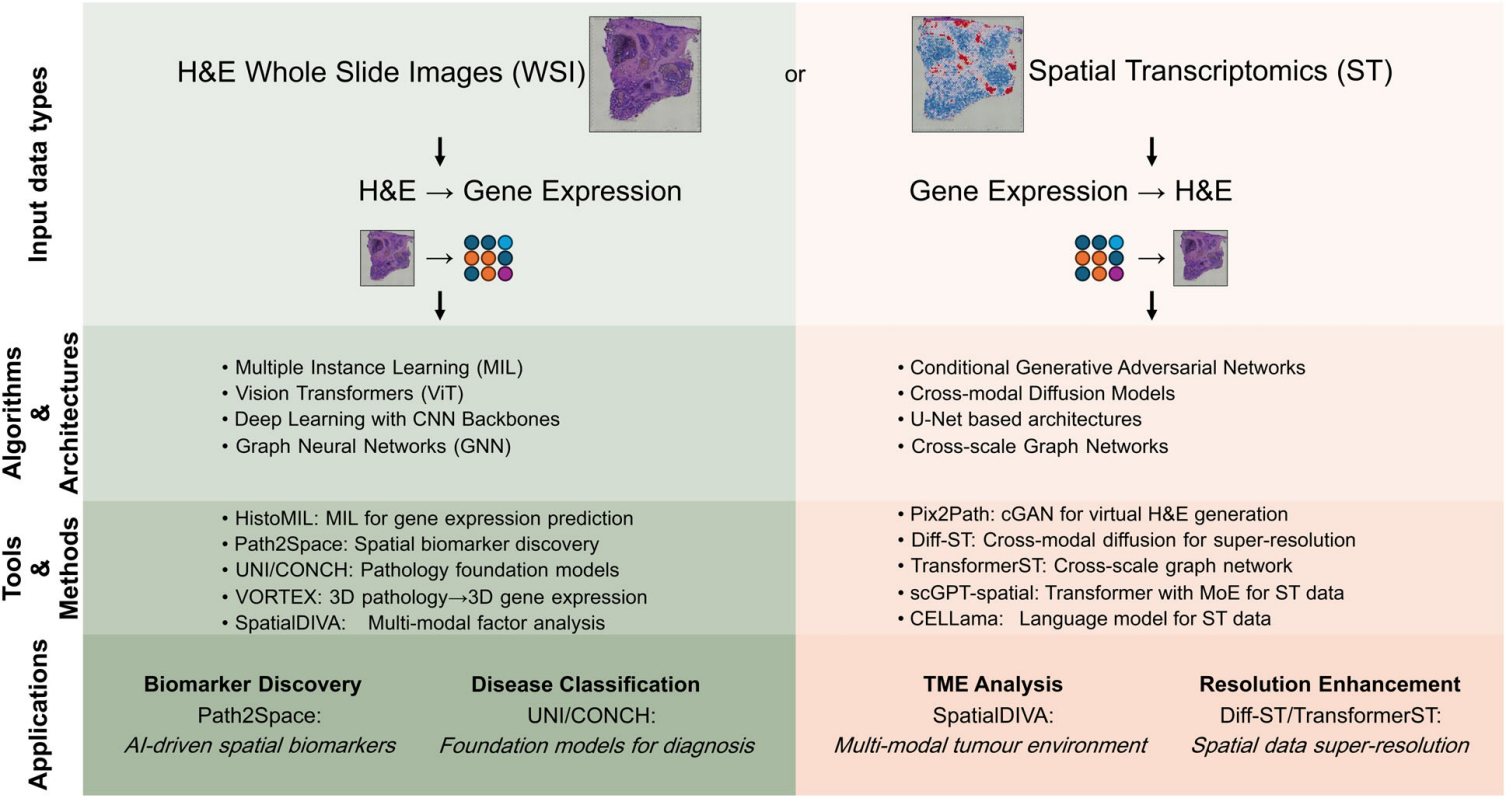
Integrating digital pathology with matched gene expression profiles. A summary of methods to integrate spatial transcriptomics and digital pathology, and their applications. cGAN, conditional Generative Adversarial Network; H&E, haematoxylin and eosin; MoE, mixture of experts; ST, spatial transcriptomics; WSI, whole slide images.

Beyond applications to ST data analysis, AI has proven to have remarkable power in capturing biologically relevant patterns in another spatially resolved arena, that of digital pathology [115, 116]. Early studies in the field successfully integrated bulk DNA or RNA sequencing with haematoxylin & eosin (H&E) stained slides to predict various clinically relevant features in histopathology images, including microsatellite instability [117], genetic alterations [118], gene expression or cancer prognosis [119]. While still in its infancy and with far more modest results to date, the integration of ST with digital pathology through AI is well poised to achieve highly spatially resolved predictions, establishing bidirectional analytical frameworks that exploit the inherent multimodal nature of ST data. This integration operates along two principal axes: image‐centric approaches that predict spatially resolved molecular information from histopathology, and transcriptomics‐centric methods that enhance spatial genomic data with morphological context (Fig. 3, Table 4).

**Table 4:** Existing AI methods that integrate ST and digital pathology. Tools are listed in alphabetical order.

Tool	Method	Design goal	References
CELLama	Sentence transformer (language model)	Adapt language models for scRNA‐seq and ST analysis	Choi et al. [120]
CONCH	Vision transformer (ViT)	Pathology and language foundation model (integrating image and language)	Lu et al. [121]
Diff‐ST	Cross‐modal conditional diffusion model	Super‐resolve ST maps using histology	Wang et al. [122]
EGNv2	Graph neural network	Predict gene expression from histology slides	Yang et al. [123]
HistoGene	Vision transformer (ViT)	Predict gene expression from histology slides	Pang et al. [124]
iStar	Vision transformer	Integrate ST data and high‐resolution histology images to predict spatial gene expression with super‐resolution	Zhang et al. [125]
Path2Space	Deep learning	Predict spatial gene expression from histology for biomarker discovery	Shulman et al. [126]
PEKA	Parameter‐efficient knowledge transfer	Modality alignment	Pan et al. [127]
Pix2Path	Conditional Generative Adversarial Networks (cGANs), U‐Net	Infer virtual digital pathology images from ST data	Fu et al. [128]
PRESENT	Multi‐view autoencoder, graph attention network (GAT), Bayesian neural network (BNN)	Cross‐modal representation & multi‐sample integration for spatial omics data	Lv et al. [129]
scGPT‐spatial	Transformer, mixture‐of‐experts (MoE), graph neural network (GNN)	Foundation model for ST (adapting single‐cell FM)	Wang et al. [130]
SEDR	Unsupervised representation learning with variational graph autoencoders	Generate dimensionally reduced representations that capture both expression patterns and spatial context	Xu et al. [70]
SEPAL	Graph neural network	Predict gene expression from histology slides	Mejia et al. [131]
SPACE	Cell–cell interaction‐aware general framework	Reveal spatially defined cell subtypes and distinct tissue modules with characteristic intercellular signalling networks	Li et al. [114]
SpatialDIVA	Deep latent variable model (VAE‐based)	Multimodal decoupling of ST and histopathology data	Maan et al. [132]
SPELL	Chain‐of‐thought	Spatial Prompt‐Enhanced Zero‐Shot Learning for spatial aware common embeddings	https://openreview.net/forum?id=Sh88LK85AR
STAIG	Graph‐contrastive learning	Alignment‐free integration method	Yang et al. [95]
THREADS	Vision transformer	Capture the tissue's underlying molecular composition, yielding powerful representations applicable to a wide array of downstream tasks	Vaidya et al. [133]
TransformerST	Cross‐scale graph network, transformer	Enhance ST resolution & reveal structure–function links	Zhao et al. [134]
TriPath	3DCNN and 3D vision transformers	A 3D pathology deep learning platform for clinical endpoint prediction	Song et al. [135]
UNI	Vision transformer (ViT)	General‐purpose pathology foundation model (disease detection, diagnosis, prognosis)	Chen et al. [136]
UPSST	Graph attention neural network (GAT)	Integrate tissue morphology, impute gene expression and cluster spatial regions	Chen et al. [137]
VORTEX	Deep learning	Predict volumetric 3D ST from 3D pathology	Almagro‐Perez et al. [138]

Histopathology foundation models, including Phikon‐v2 [139], UNI [136] and CONCH [121], originally developed as powerful feature extractors from whole slide images, are increasingly being adapted to predict treatment outcomes and gene expression patterns directly from H&E‐stained sections [133, 140]. These models demonstrate superior performance compared with earlier approaches developed by us [141] and others [142, 143], which employed multi‐instance learning to predict multiple biomarkers from histological images. However, despite their promise as economical alternatives to costly ST experiments, these prediction methods face inherent limitations—primarily that only expression changes manifesting as morphological alterations can be detected in H&E slides, creating a natural ceiling for prediction accuracy. A way to bypass this for clinical benefit is focusing on pathways or tissue‐level processes which may be more easily predicted than individual gene activity.

While cutting‐edge ST technologies can achieve single‐cell resolution, many widely available datasets still utilise earlier‐generation platforms with spot‐level resolution containing multiple cells, creating a practical constraint for training comprehensive AI models at true single‐cell or subcellular scales. This technological constraint has driven the development of AI‐based super‐resolution approaches that leverage high‐resolution histological images to enhance transcriptomic data interpretation. Diff‐ST [144] exemplifies this strategy as a cross‐modal conditional diffusion model that addresses key challenges in ST super‐resolution, such as restoration uncertainty and mode collapse, while TransformerST [134] utilises vision transformer encoders and cross‐scale graph networks to achieve single‐cell granularity from multicellular data, and iStar [125] leverages hierarchical image feature extraction to predict gene expression at near‐single‐cell resolution, even in tissue sections with only histology images available. Beyond resolution enhancement, integrative frameworks like UPSST [137] combine tissue morphology with ST through graph attention networks for unsupervised pathology domain identification, achieving high accuracy in delineating diseased regions.

Path2Space [126] demonstrates the potential clinical impact of these integration methods by successfully identifying novel spatially localised breast cancer subgroups with distinct survival outcomes through large‐scale biomarker discovery using existing pathology archives. Levy‐Jurgenson et al. [145] spatially resolve transcriptional profiles from pathology images and use these to develop a heterogeneity index linked with survival in breast and lung cancers.

Three‐dimensional integration represents another significant advancement in this domain, with VORTEX [138] enabling volumetric prediction of ST from 3D pathological datasets through learned morpho‐molecular associations, providing unprecedented insights into tissue architecture and microenvironmental complexity otherwise unattainable through traditional 2D analysis (see also tools like TriPath [135] or OpenST [146]). Additionally, recent advances, such as spaVAE and its extension spaPeakVAE [147], demonstrate AI's capacity to characterise spatial ATAC‐seq data, capturing the complex spatial dependencies in chromatin accessibility patterns that complement transcriptomic information.

Recent developments increasingly blur boundaries between these paradigms through truly integrative approaches. PRESENT [148] advances cross‐modality representation learning for spatial omics data integration across multiple samples and technologies, while SpatialDIVA [132] addresses the critical challenge of deconvolving shared and distinct sources of variation between ST and histopathology data, separating factors, including expression variability, spatial context, tissue morphology and batch effects. Meanwhile, the adaptation of single‐cell foundation models for spatial analysis continues to gain traction, with scGPT‐spatial [130] demonstrating continuous pretraining strategies on spatial datasets, and CELLama [120] transforming spatial data into language‐compatible formats suitable for pretrained language models. SPELL (https://openreview.net/pdf?id=Sh88LK85AR) leverages chain‐of‐thought prompting alongside graph autoencoder‐derived spatial embeddings for zero‐shot cell‐type classification. This approach highlights the critical role of spatial context in improving model performance. Our method, PEKA [127], employs parameter‐efficient knowledge transfer with block‐affine adaptation to enhance gene expression prediction from histopathology images, achieving significant improvements over baseline foundation models.

Resource development remains crucial to this field's advancement [149] with datasets like HEST‐1k [150] providing over 1000 paired ST maps and whole slide images for benchmarking and model development. Despite remarkable progress, significant challenges persist: (a) scale disparities between morphological features and molecular signals, (b) data heterogeneity across platforms and (c) complex AI model interpretability requirements. Standardisation of protocols and data formats represents an essential prerequisite for developing robust, generalisable models capable of integrating diverse ST technologies with digital pathology across varying resolutions. As these challenges are systematically addressed, the integration of ST with digital pathology holds transformative potential for advancing biological understanding and augmenting clinical diagnostics.

## 6 Moving towards clinical implementation

Either through integration with digital pathology or as a standalone tool to uncover spatial organisation and markers that may inform diagnosis or treatment, ST is well poised to aid clinical decision workflows in the future [151, 152]. To date, ST has been used in cancer research to discover biomarkers for disease onset or response to therapy [153, 154, 155], and to disentangle complex molecular networks of alterations both spatially and temporally [156, 157].

ST can enable clinical translation by elucidating key mechanisms driving cancer progression and treatment resistance, often through niche remodelling and specific TME interactions [158, 159, 160, 161]. A pertinent example of how spatial profiling of tumours can uncover novel biomarkers of therapeutic response is provided by Acha‐Sagredo et al. [162], who discovered that the MHC class II invariant chain CD74 is specifically upregulated in cancer cells and tumour‐associated macrophages near T cells in responders to immune checkpoint blockade in mismatch repair proficient colorectal cancer. ST can also decipher the heterogeneity and spatial compartmentalisation at the core and leading edge of tumours [163], which could provide an opportunity to revisit patient stratification strategies and inform adjuvant therapy options. It can also be leveraged to understand cell competition within the tumour niche [164, 165, 166], paving the way towards predicting resistant clone expansions upon therapy. Recently, the utility of profiling non‐coding RNAs in ST data was demonstrated for early detection of metastatic disease in colorectal cancer [167].

Furthermore, an increasing number of studies are applying ST to directly investigate the effect of selected treatment regimes. For instance, Shiao et al. [168] have compared spatially profiled cancer samples before and after pembrolizumab and radiotherapy, defining spatial signatures of response to treatment. Lengrand et al. [169] have exploited ST datasets to demonstrate a spatial reduction in EMT upon inhibition with Netrin‐1 in endometrial cancer, while Rubinstein et al. [157] have characterised persister cell dynamics after targeted treatment in melanoma.

The advent of ST has also highlighted that classical cancer subtyping, for example as performed in breast [170], colorectal [171] or brain cancers [172] during the bulk sequencing era, fails to comprehensively capture the heterogeneity of most tumour tissues. In fact, spatial profiling is increasingly revealing that many tumours comprise distinct regions of subtype‐specific nature [173, 174] with divergent molecular mechanisms. Thus, elucidating the distribution of niches with a distinct molecular profile using ST provides an opportunity to understand targeting opportunities in a spatially resolved manner.

Despite the promising potential of ST in preclinical research and its unprecedented resolution allowing mechanistic insights, its translation into routine clinical practice is challenging due to the high costs, delicate nature of library preparation and time‐intensive data preprocessing, annotation and analysis [175]. Furthermore, there is a need for better standardised protocols, high‐quality reference datasets and benchmarks for validating spatial predictions. Thus, the most immediate clinical impact of ST is likely to come from its capacity to uncover novel mechanisms that can be exploited for therapeutic benefit, particularly in rationalising combination therapies. Another promising avenue lies in leveraging ST datasets to train AI models for refined patient stratification and personalised treatment strategies. Approaches aiming to define cellular niches (rather than just cells) that can respond to different therapies could revolutionise treatment strategies and are also more likely to show morphological changes that can be captured in histology slides. The success of digital pathology in augmenting pathological diagnosis has already been demonstrated by tools like CellProfiler [176] or QuPath [177, 178], and ST could further potentiate these readouts. If digital pathology and ST are successfully integrated, advanced AI models could be employed alongside routine histopathology analysis to provide predictions for survival, biomarkers or recommended treatment routes that take into account tumour heterogeneity.

Naturally, the applications of ST extend beyond cancer to other diseases – for instance, illustrating spatiotemporal dynamics of gene expression during the progression of amyotrophic lateral sclerosis [179]. We envision that similar longitudinal applications will be crucial in understanding cancer pathogenesis from preneoplasia to advanced disease. For additional insights into the challenges and opportunities of translating spatial technologies into clinical practice, Pentimalli et al. [180] offer a valuable complementary perspective.

## 7 Conclusion and future perspectives

ST represents a transformative leap in transcriptomic profiling, bridging the gap between molecular data and tissue architecture. As the field continues to evolve, it is crucial to adopt rigorous data preprocessing strategies (i.e. quality control, normalisation and batch correction) and establish benchmarking protocols to ensure reproducibility and biological fidelity. Integrating spatial data with complementary modalities, such as imaging, metabolomics or proteomics will further enrich our understanding of complex tissue ecosystems, particularly in the context of the TME. Going beyond simply identifying niches to characterising their spatial distribution and assessing whether this distribution is biologically or clinically relevant, alongside identifying cellular vulnerabilities within cancer niches *in situ*, is key to moving the field forward.

While most studies identify correlations between cell populations, applying causal reasoning or similar statistical methods will be key to defining causal relationships, thereby opening up opportunities to elucidate system‐wide dependencies. Furthermore, while the current focus is mostly on relatively short‐range interactions, defining and exploring longer‐range interactions and emerging properties from the broader spatial organisation of the tissue will likely yield novel biology much beyond what has already been characterised in *in vitro* and *in vivo* studies. We envision that geospatial and AI methods may play an important role in this respect, but they must be applied with caution, as biological systems involve complexities not present in the settings where these tools have been originally developed. A key emerging frontier is the expansion from 2D to 3D and the integration of multi‐omics modalities spatially to understand tissue organisation [146, 181, 182, 183]—ideally combined with modelling approaches to create virtual cancer avatars [184] that can be used for AI‐enabled predictions and drug prioritisation.

Finally, when it comes to bringing ST insights into the clinic, two paths are laid out: one is through classical mechanistic understanding of cellular relationships and vulnerabilities and how they can be targeted within defined niches; the other is through facilitation of diagnosis and treatment management by augmenting digital pathology readouts. Successful prediction of individual biomarkers from digital pathology may be limited to a handful of clinically relevant proteins with roles affecting cellular morphology. However, predicting cell behaviour at the broader scale of ‘functional’ cellular niches is likely an achievable task, and this may pave the way to cost‐effective AI‐aided clinical decisions. Thus, with ongoing technological and computational advancements, ST stands valid to uncover previously inaccessible layers of biological insights and drive the next generation of discoveries in tumour ecosystems.

## Conflict of interest

The authors declare no conflict of interest.

## Author contributions

MS conceptualised and structured the study, and provided input to each section. CC discussed current analysis workflows, methods to identify cellular niches and dissect cell–cell communication, designed Figs 1 and 2 and provided additional input to all other sections. SP discussed the integration of AI with digital pathology and designed Fig. 3. EW discussed the identification of cell states, geospatial and AI approaches to defining a cellular niche. HWL discussed the clinical applications of ST together with MS. All authors read and approved the manuscript.
